# Bis[2-(cyclo­pentyl­imino­meth­yl)-5-meth­oxy­phenolato]copper(II)

**DOI:** 10.1107/S1600536810025481

**Published:** 2010-07-03

**Authors:** Xiao-Hui Ji, Jiu-Fu Lu

**Affiliations:** a School of Chemistry and Environmental Science, Shaanxi University of Technology, Hanzhong 723000, People’s Republic of China

## Abstract

The title compound, [Cu(C_13_H_16_NO_2_)_2_], is a mononuclear copper(II) complex derived from the Schiff base ligand 2-(cyclo­pentyl­imino­meth­yl)-5-meth­oxy­phenol and copper acetate. The Cu^II^ atom is four-coordinated by the phenolate O atoms and imine N atoms from two Schiff base ligands, in a highly distorted square-planar geometry. The O- and N-donor atoms are mutually *trans* and the dihedral angle between the two benzene rings is 55.8 (3)°.

## Related literature

For background to complexes with Schiff bases, see: Hamaker *et al.* (2010 ▶); Wang *et al.* (2010 ▶); Mirkhani *et al.* (2010 ▶); Liu & Yang (2009 ▶); Keypour *et al.* (2009 ▶); Adhikary *et al.* (2009 ▶); Peng *et al.* (2009 ▶). For similar copper complexes, see: Friščić *et al.* (2002 ▶); Marsh & Spek (2001 ▶); Han *et al.* (2001 ▶); Akitsu & Einaga (2004 ▶); Dhar *et al.* (2003 ▶).
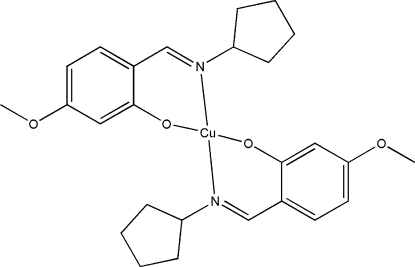


## Experimental

### 

#### Crystal data

[Cu(C_13_H_16_NO_2_)_2_]
*M* *_r_* = 500.08
Monoclinic, 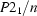

*a* = 8.496 (1) Å
*b* = 14.054 (2) Å
*c* = 20.442 (2) Å
β = 100.236 (3)°
*V* = 2402.0 (5) Å^3^
*Z* = 4
Mo *K*α radiation
μ = 0.94 mm^−1^
*T* = 298 K
0.23 × 0.21 × 0.21 mm

#### Data collection

Bruker APEXII CCD area-detector diffractometer
Absorption correction: multi-scan (*SADABS*; Sheldrick, 2004 ▶) *T*_min_ = 0.812, *T*_max_ = 0.826
12222 measured reflections
4333 independent reflections
3131 reflections with *I* > 2σ(*I*)
*R*_int_ = 0.081

#### Refinement

*R*[*F* ^2^ > 2σ(*F* ^2^)] = 0.057
*wR*(*F* ^2^) = 0.163
*S* = 1.00
4333 reflections
300 parameters
H-atom parameters constrained
Δρ_max_ = 0.69 e Å^−3^
Δρ_min_ = −1.16 e Å^−3^

### 

Data collection: *APEX2* (Bruker, 2004 ▶); cell refinement: *SAINT* (Bruker, 2004 ▶); data reduction: *SAINT*; program(s) used to solve structure: *SHELXS97* (Sheldrick, 2008 ▶); program(s) used to refine structure: *SHELXL97* (Sheldrick, 2008 ▶); molecular graphics: *SHELXTL* (Sheldrick, 2008 ▶); software used to prepare material for publication: *SHELXTL*.

## Supplementary Material

Crystal structure: contains datablocks global, I. DOI: 10.1107/S1600536810025481/sj5030sup1.cif

Structure factors: contains datablocks I. DOI: 10.1107/S1600536810025481/sj5030Isup2.hkl

Additional supplementary materials:  crystallographic information; 3D view; checkCIF report

## Figures and Tables

**Table:** 

Cu1—O1	1.890 (2)
Cu1—O3	1.891 (2)
Cu1—N2	1.967 (3)
Cu1—N1	1.978 (3)

**Table:** 

O1—Cu1—O3	144.60 (13)
O1—Cu1—N2	93.93 (11)
O3—Cu1—N2	95.32 (11)
O1—Cu1—N1	95.40 (11)
O3—Cu1—N1	94.20 (11)
N2—Cu1—N1	148.66 (12)

**Fig. 1.**
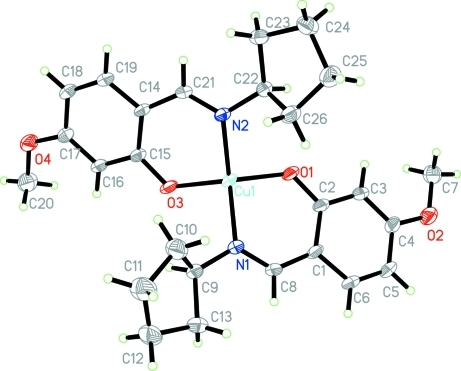
The molecular structure of the title complex, showing 30% probability displacement ellipsoids and the atom-numbering scheme.

## supplementary crystallographic information

### Comment

Schiff bases are known to be versatile ligands in coordination chemistry (Hamaker *et al.*, 2010; Wang *et al.*, 2010; Mirkhani *et al.*, 2010; Liu & Yang, 2009). A large number of complexes with Schiff bases have been reported because of their interesting structures and potential applications (Keypour *et al.*, 2009; Adhikary *et al.*, 2009; Peng *et al.*, 2009). We report here the crystal structure of the title new copper complex with the Schiff base ligand 2-(cyclopentyliminomethyl)-5-methoxyphenol.

The Cu atom in the complex is four-coordinated by two phenolate O atoms and two imine N atoms from two Schiff base ligands, forming a distorted square planar geometry (Fig. 1). The dihedral angle between the C1-C6 and C14-C19 benzene rings is 55.8 (3)°. The bond lengths (Table 1) involving the Cu atom are comparable to those observed in similar copper complexes (Friščić *et al.*, 2002; Marsh & Spek, 2001; Han *et al.*, 2001; Akitsu & Einaga, 2004; Dhar *et al.*, 2003).

### Experimental

4-Methoxysalicylaldehyde (0.1 mmol, 15.2 mg) and cyclopentylamine (0.1 mmol, 8.5 mg) were mixed and stirred in methanol (10 ml) for 30 min. Then a methanol solution (5 ml) of copper acetate (0.1 mmol, 19.9 mg) was added to the mixture. The final mixture was stirred for another 30 min to give a blue solution. Single crystals suitable for X-ray diffraction were obtained by slow evaporation of the solution at room temperature.

### Refinement

H atoms were positioned geometrically (C—H = 0.93–0.97 Å) and refined using a riding model, with with *U*_iso_(H) = 1.2*U*_eq_(C) and 1.5*U*_eq_(C_metyl_). Rotating group models were used for the methyl groups.

### Crystal data

**Table:**

[Cu(C_13_H_16_NO_2_)_2_]	*F*(000) = 1052
*M**_r_* = 500.08	*D*_x_ = 1.383 Mg m^−^^3^
Monoclinic, *P*2_1_/*n*	Mo *K*α radiation, λ = 0.71073 Å
Hall symbol: -P 2yn	Cell parameters from 3695 reflections
*a* = 8.496 (1) Å	θ = 2.5–25.1°
*b* = 14.054 (2) Å	µ = 0.94 mm^−^^1^
*c* = 20.442 (2) Å	*T* = 298 K
β = 100.236 (3)°	Block, blue
*V* = 2402.0 (5) Å^3^	0.23 × 0.21 × 0.21 mm
*Z* = 4	

### Data collection

**Table:**

Bruker APEXII CCD area-detector diffractometer	4333 independent reflections
Radiation source: fine-focus sealed tube	3131 reflections with *I* > 2σ(*I*)
graphite	*R*_int_ = 0.081
ω scans	θ_max_ = 25.3°, θ_min_ = 1.8°
Absorption correction: multi-scan (*SADABS*; Sheldrick, 2004)	*h* = −9→10
*T*_min_ = 0.812, *T*_max_ = 0.826	*k* = −16→16
12222 measured reflections	*l* = −24→17

### Refinement

**Table:**

Refinement on *F*^2^	Primary atom site location: structure-invariant direct methods
Least-squares matrix: full	Secondary atom site location: difference Fourier map
*R*[*F*^2^ > 2σ(*F*^2^)] = 0.057	Hydrogen site location: inferred from neighbouring sites
*wR*(*F*^2^) = 0.163	H-atom parameters constrained
*S* = 1.00	*w* = 1/[σ^2^(*F*_o_^2^) + (0.0973*P*)^2^] where *P* = (*F*_o_^2^ + 2*F*_c_^2^)/3
4333 reflections	(Δ/σ)_max_ = 0.001
300 parameters	Δρ_max_ = 0.69 e Å^−^^3^
0 restraints	Δρ_min_ = −1.16 e Å^−^^3^

### Special details

**Table:**

Geometry. All esds (except the esd in the dihedral angle between two l.s. planes) are estimated using the full covariance matrix. The cell esds are taken into account individually in the estimation of esds in distances, angles and torsion angles; correlations between esds in cell parameters are only used when they are defined by crystal symmetry. An approximate (isotropic) treatment of cell esds is used for estimating esds involving l.s. planes.
Refinement. Refinement of F^2^ against ALL reflections. The weighted R-factor wR and goodness of fit S are based on F^2^, conventional R-factors R are based on F, with F set to zero for negative F^2^. The threshold expression of F^2^ > 2sigma(F^2^) is used only for calculating R-factors(gt) etc. and is not relevant to the choice of reflections for refinement. R-factors based on F^2^ are statistically about twice as large as those based on F, and R- factors based on ALL data will be even larger.

### Fractional atomic coordinates and isotropic or equivalent isotropic displacement parameters (Å^2^)

**Table:**

	*x*	*y*	*z*	*U*_iso_*/*U*_eq_	
Cu1	−0.00311 (5)	0.84349 (3)	0.75910 (2)	0.0392 (2)	
N1	−0.0266 (3)	0.8673 (2)	0.85224 (14)	0.0387 (7)	
N2	0.0215 (3)	0.8950 (2)	0.67202 (13)	0.0367 (7)	
O1	0.2109 (3)	0.80068 (19)	0.77995 (11)	0.0460 (6)	
O2	0.6781 (3)	0.6732 (2)	0.91142 (15)	0.0597 (8)	
O3	−0.2181 (3)	0.8050 (2)	0.73199 (11)	0.0487 (7)	
O4	−0.6953 (3)	0.7268 (2)	0.58341 (13)	0.0608 (8)	
C1	0.2376 (4)	0.8058 (3)	0.89943 (16)	0.0387 (8)	
C2	0.2940 (4)	0.7847 (2)	0.83963 (16)	0.0376 (8)	
C3	0.4436 (4)	0.7414 (2)	0.84350 (17)	0.0408 (8)	
H3	0.4817	0.7276	0.8047	0.049*	
C4	0.5355 (4)	0.7190 (3)	0.90438 (18)	0.0438 (9)	
C5	0.4826 (5)	0.7423 (3)	0.96339 (18)	0.0510 (10)	
H5	0.5457	0.7289	1.0044	0.061*	
C6	0.3390 (4)	0.7843 (3)	0.96003 (18)	0.0481 (10)	
H6	0.3051	0.7999	0.9995	0.058*	
C7	0.7322 (5)	0.6397 (3)	0.8541 (2)	0.0614 (12)	
H7A	0.6525	0.5990	0.8293	0.092*	
H7B	0.8297	0.6046	0.8671	0.092*	
H7C	0.7513	0.6928	0.8270	0.092*	
C8	0.0844 (4)	0.8467 (2)	0.90186 (18)	0.0404 (9)	
H8	0.0625	0.8599	0.9439	0.049*	
C9	−0.1767 (4)	0.9093 (3)	0.86370 (17)	0.0414 (8)	
H9	−0.2630	0.8665	0.8436	0.050*	
C10	−0.2091 (5)	1.0059 (3)	0.8299 (2)	0.0653 (12)	
H10A	−0.1117	1.0433	0.8349	0.078*	
H10B	−0.2508	0.9980	0.7828	0.078*	
C11	−0.3317 (6)	1.0539 (4)	0.8645 (3)	0.0819 (16)	
H11A	−0.2954	1.1169	0.8796	0.098*	
H11B	−0.4332	1.0599	0.8344	0.098*	
C12	−0.3499 (5)	0.9915 (3)	0.9232 (2)	0.0670 (13)	
H12A	−0.4458	0.9529	0.9131	0.080*	
H12B	−0.3555	1.0298	0.9622	0.080*	
C13	−0.2011 (5)	0.9294 (3)	0.9345 (2)	0.0563 (11)	
H13A	−0.2186	0.8711	0.9575	0.068*	
H13B	−0.1102	0.9629	0.9596	0.068*	
C14	−0.2496 (4)	0.8557 (2)	0.61796 (17)	0.0393 (9)	
C15	−0.3019 (4)	0.8089 (3)	0.67175 (17)	0.0390 (8)	
C16	−0.4528 (4)	0.7646 (3)	0.66019 (17)	0.0432 (9)	
H16	−0.4880	0.7324	0.6947	0.052*	
C17	−0.5492 (4)	0.7683 (3)	0.59848 (18)	0.0448 (9)	
C18	−0.4993 (5)	0.8175 (3)	0.54602 (19)	0.0540 (11)	
H18	−0.5656	0.8213	0.5047	0.065*	
C19	−0.3540 (4)	0.8592 (3)	0.55627 (18)	0.0497 (10)	
H19	−0.3216	0.8916	0.5213	0.060*	
C20	−0.7465 (6)	0.6658 (3)	0.6315 (3)	0.0699 (14)	
H20A	−0.6642	0.6202	0.6469	0.105*	
H20B	−0.8423	0.6332	0.6115	0.105*	
H20C	−0.7675	0.7032	0.6683	0.105*	
C21	−0.0928 (4)	0.8941 (2)	0.62103 (17)	0.0400 (8)	
H21	−0.0707	0.9215	0.5822	0.048*	
C22	0.1781 (4)	0.9340 (2)	0.66504 (17)	0.0386 (8)	
H22	0.2540	0.8808	0.6702	0.046*	
C23	0.1945 (4)	0.9857 (3)	0.60076 (18)	0.0472 (9)	
H23A	0.1985	0.9408	0.5651	0.057*	
H23B	0.1057	1.0290	0.5873	0.057*	
C24	0.3521 (5)	1.0401 (3)	0.6185 (2)	0.0557 (11)	
H24A	0.3421	1.1032	0.5989	0.067*	
H24B	0.4372	1.0065	0.6022	0.067*	
C25	0.3882 (5)	1.0467 (3)	0.6946 (2)	0.0631 (12)	
H25A	0.4823	1.0096	0.7124	0.076*	
H25B	0.4067	1.1123	0.7087	0.076*	
C26	0.2415 (5)	1.0072 (3)	0.71846 (19)	0.0493 (10)	
H26A	0.1632	1.0567	0.7208	0.059*	
H26B	0.2703	0.9774	0.7617	0.059*	

### Atomic displacement parameters (Å^2^)

**Table:**

	*U*^11^	*U*^22^	*U*^33^	*U*^12^	*U*^13^	*U*^23^
Cu1	0.0408 (3)	0.0561 (3)	0.0189 (3)	0.00078 (18)	0.00073 (19)	0.00373 (18)
N1	0.0382 (17)	0.0517 (17)	0.0256 (16)	−0.0011 (13)	0.0040 (13)	0.0007 (13)
N2	0.0390 (16)	0.0482 (17)	0.0227 (15)	0.0050 (13)	0.0045 (12)	0.0025 (13)
O1	0.0477 (15)	0.0664 (16)	0.0218 (13)	0.0132 (13)	0.0001 (11)	0.0091 (12)
O2	0.0506 (17)	0.082 (2)	0.0432 (17)	0.0161 (14)	−0.0017 (14)	0.0044 (15)
O3	0.0483 (15)	0.0762 (17)	0.0189 (13)	−0.0110 (13)	−0.0013 (11)	0.0067 (12)
O4	0.0481 (17)	0.093 (2)	0.0370 (16)	−0.0184 (15)	−0.0033 (13)	−0.0022 (15)
C1	0.044 (2)	0.049 (2)	0.0206 (18)	0.0027 (16)	−0.0030 (15)	−0.0013 (16)
C2	0.047 (2)	0.0435 (19)	0.0215 (17)	−0.0014 (16)	0.0030 (15)	0.0049 (15)
C3	0.045 (2)	0.052 (2)	0.0262 (18)	0.0029 (17)	0.0067 (16)	0.0036 (16)
C4	0.042 (2)	0.050 (2)	0.036 (2)	0.0022 (16)	−0.0022 (16)	0.0066 (17)
C5	0.051 (2)	0.070 (3)	0.027 (2)	0.0025 (19)	−0.0074 (17)	0.0035 (19)
C6	0.055 (2)	0.067 (3)	0.0202 (18)	0.0014 (19)	0.0018 (17)	−0.0019 (18)
C7	0.053 (3)	0.071 (3)	0.061 (3)	0.019 (2)	0.012 (2)	0.009 (2)
C8	0.044 (2)	0.055 (2)	0.0227 (18)	−0.0036 (16)	0.0081 (16)	−0.0031 (16)
C9	0.040 (2)	0.055 (2)	0.0281 (19)	−0.0057 (16)	0.0050 (15)	−0.0045 (17)
C10	0.062 (3)	0.073 (3)	0.064 (3)	0.018 (2)	0.021 (2)	0.018 (2)
C11	0.092 (4)	0.082 (3)	0.076 (4)	0.032 (3)	0.028 (3)	0.010 (3)
C12	0.070 (3)	0.073 (3)	0.063 (3)	0.009 (2)	0.024 (2)	−0.017 (2)
C13	0.061 (3)	0.072 (3)	0.038 (2)	0.005 (2)	0.017 (2)	−0.005 (2)
C14	0.042 (2)	0.056 (2)	0.0185 (18)	0.0033 (16)	0.0012 (15)	0.0013 (15)
C15	0.043 (2)	0.051 (2)	0.0215 (18)	0.0045 (16)	0.0028 (15)	−0.0023 (16)
C16	0.044 (2)	0.061 (2)	0.0234 (18)	−0.0042 (17)	0.0024 (16)	0.0031 (17)
C17	0.044 (2)	0.060 (2)	0.0279 (19)	−0.0007 (17)	0.0004 (16)	−0.0064 (18)
C18	0.049 (2)	0.086 (3)	0.023 (2)	−0.002 (2)	−0.0044 (17)	0.001 (2)
C19	0.048 (2)	0.077 (3)	0.023 (2)	−0.0010 (19)	0.0031 (17)	0.0059 (18)
C20	0.061 (3)	0.087 (3)	0.060 (3)	−0.026 (2)	0.005 (2)	0.003 (3)
C21	0.044 (2)	0.054 (2)	0.0228 (18)	0.0017 (17)	0.0071 (15)	0.0070 (16)
C22	0.038 (2)	0.045 (2)	0.0319 (19)	0.0040 (15)	0.0053 (15)	0.0026 (16)
C23	0.053 (2)	0.056 (2)	0.034 (2)	−0.0034 (18)	0.0110 (18)	0.0057 (18)
C24	0.059 (3)	0.058 (2)	0.053 (3)	−0.013 (2)	0.020 (2)	−0.002 (2)
C25	0.065 (3)	0.068 (3)	0.053 (3)	−0.018 (2)	0.000 (2)	0.005 (2)
C26	0.057 (2)	0.056 (2)	0.034 (2)	−0.0040 (18)	0.0031 (18)	−0.0020 (18)

### Geometric parameters (Å, °)

**Table:**

Cu1—O1	1.890 (2)	C11—H11A	0.9700
Cu1—O3	1.891 (2)	C11—H11B	0.9700
Cu1—N2	1.967 (3)	C12—C13	1.520 (6)
Cu1—N1	1.978 (3)	C12—H12A	0.9700
N1—C8	1.289 (4)	C12—H12B	0.9700
N1—C9	1.462 (4)	C13—H13A	0.9700
N2—C21	1.292 (4)	C13—H13B	0.9700
N2—C22	1.469 (4)	C14—C19	1.408 (5)
O1—C2	1.316 (4)	C14—C15	1.419 (5)
O2—C4	1.356 (4)	C14—C21	1.428 (5)
O2—C7	1.414 (5)	C15—C16	1.407 (5)
O3—C15	1.309 (4)	C16—C17	1.378 (5)
O4—C17	1.356 (4)	C16—H16	0.9300
O4—C20	1.429 (5)	C17—C18	1.403 (5)
C1—C6	1.410 (5)	C18—C19	1.349 (5)
C1—C2	1.421 (5)	C18—H18	0.9300
C1—C8	1.432 (5)	C19—H19	0.9300
C2—C3	1.398 (5)	C20—H20A	0.9600
C3—C4	1.383 (5)	C20—H20B	0.9600
C3—H3	0.9300	C20—H20C	0.9600
C4—C5	1.399 (5)	C21—H21	0.9300
C5—C6	1.346 (5)	C22—C26	1.527 (5)
C5—H5	0.9300	C22—C23	1.529 (5)
C6—H6	0.9300	C22—H22	0.9800
C7—H7A	0.9600	C23—C24	1.529 (5)
C7—H7B	0.9600	C23—H23A	0.9700
C7—H7C	0.9600	C23—H23B	0.9700
C8—H8	0.9300	C24—C25	1.535 (6)
C9—C13	1.524 (5)	C24—H24A	0.9700
C9—C10	1.527 (5)	C24—H24B	0.9700
C9—H9	0.9800	C25—C26	1.522 (5)
C10—C11	1.518 (6)	C25—H25A	0.9700
C10—H10A	0.9700	C25—H25B	0.9700
C10—H10B	0.9700	C26—H26A	0.9700
C11—C12	1.516 (6)	C26—H26B	0.9700
			
O1—Cu1—O3	144.60 (13)	C13—C12—H12B	110.8
O1—Cu1—N2	93.93 (11)	H12A—C12—H12B	108.9
O3—Cu1—N2	95.32 (11)	C12—C13—C9	102.3 (3)
O1—Cu1—N1	95.40 (11)	C12—C13—H13A	111.3
O3—Cu1—N1	94.20 (11)	C9—C13—H13A	111.3
N2—Cu1—N1	148.66 (12)	C12—C13—H13B	111.3
C8—N1—C9	120.1 (3)	C9—C13—H13B	111.3
C8—N1—Cu1	122.4 (2)	H13A—C13—H13B	109.2
C9—N1—Cu1	117.6 (2)	C19—C14—C15	118.5 (3)
C21—N2—C22	119.3 (3)	C19—C14—C21	117.4 (3)
C21—N2—Cu1	122.7 (2)	C15—C14—C21	124.0 (3)
C22—N2—Cu1	118.0 (2)	O3—C15—C16	117.8 (3)
C2—O1—Cu1	126.8 (2)	O3—C15—C14	123.7 (3)
C4—O2—C7	119.1 (3)	C16—C15—C14	118.5 (3)
C15—O3—Cu1	126.8 (2)	C17—C16—C15	120.8 (3)
C17—O4—C20	118.7 (3)	C17—C16—H16	119.6
C6—C1—C2	117.6 (3)	C15—C16—H16	119.6
C6—C1—C8	118.2 (3)	O4—C17—C16	124.2 (3)
C2—C1—C8	124.1 (3)	O4—C17—C18	115.3 (3)
O1—C2—C3	117.4 (3)	C16—C17—C18	120.5 (3)
O1—C2—C1	123.7 (3)	C19—C18—C17	119.2 (3)
C3—C2—C1	118.9 (3)	C19—C18—H18	120.4
C4—C3—C2	120.8 (3)	C17—C18—H18	120.4
C4—C3—H3	119.6	C18—C19—C14	122.5 (4)
C2—C3—H3	119.6	C18—C19—H19	118.8
O2—C4—C3	123.7 (4)	C14—C19—H19	118.8
O2—C4—C5	115.9 (3)	O4—C20—H20A	109.5
C3—C4—C5	120.4 (3)	O4—C20—H20B	109.5
C6—C5—C4	119.1 (3)	H20A—C20—H20B	109.5
C6—C5—H5	120.5	O4—C20—H20C	109.5
C4—C5—H5	120.5	H20A—C20—H20C	109.5
C5—C6—C1	123.1 (4)	H20B—C20—H20C	109.5
C5—C6—H6	118.5	N2—C21—C14	126.9 (3)
C1—C6—H6	118.5	N2—C21—H21	116.5
O2—C7—H7A	109.5	C14—C21—H21	116.5
O2—C7—H7B	109.5	N2—C22—C26	113.1 (3)
H7A—C7—H7B	109.5	N2—C22—C23	118.9 (3)
O2—C7—H7C	109.5	C26—C22—C23	102.7 (3)
H7A—C7—H7C	109.5	N2—C22—H22	107.2
H7B—C7—H7C	109.5	C26—C22—H22	107.2
N1—C8—C1	127.2 (3)	C23—C22—H22	107.2
N1—C8—H8	116.4	C24—C23—C22	104.1 (3)
C1—C8—H8	116.4	C24—C23—H23A	110.9
N1—C9—C13	119.8 (3)	C22—C23—H23A	110.9
N1—C9—C10	112.2 (3)	C24—C23—H23B	110.9
C13—C9—C10	102.8 (3)	C22—C23—H23B	110.9
N1—C9—H9	107.1	H23A—C23—H23B	109.0
C13—C9—H9	107.1	C23—C24—C25	106.2 (3)
C10—C9—H9	107.1	C23—C24—H24A	110.5
C11—C10—C9	105.5 (3)	C25—C24—H24A	110.5
C11—C10—H10A	110.6	C23—C24—H24B	110.5
C9—C10—H10A	110.6	C25—C24—H24B	110.5
C11—C10—H10B	110.6	H24A—C24—H24B	108.7
C9—C10—H10B	110.6	C26—C25—C24	106.0 (3)
H10A—C10—H10B	108.8	C26—C25—H25A	110.5
C12—C11—C10	106.7 (4)	C24—C25—H25A	110.5
C12—C11—H11A	110.4	C26—C25—H25B	110.5
C10—C11—H11A	110.4	C24—C25—H25B	110.5
C12—C11—H11B	110.4	H25A—C25—H25B	108.7
C10—C11—H11B	110.4	C25—C26—C22	102.8 (3)
H11A—C11—H11B	108.6	C25—C26—H26A	111.2
C11—C12—C13	104.6 (3)	C22—C26—H26A	111.2
C11—C12—H12A	110.8	C25—C26—H26B	111.2
C13—C12—H12A	110.8	C22—C26—H26B	111.2
C11—C12—H12B	110.8	H26A—C26—H26B	109.1

## References

[bb1] Adhikary, C., Sen, R., Bocelli, G., Cantoni, A., Solzi, M., Chaudhuri, S. & Koner, S. (2009). *J. Coord. Chem.* **62**, 3573–3582.

[bb2] Akitsu, T. & Einaga, Y. (2004). *Acta Cryst.* E**60**, m436–m438.

[bb3] Bruker (2004). *APEX2* and *SAINT* Bruker AXS Inc., Madison, Wisconsin, USA.

[bb4] Dhar, S., Senapati, D., Das, P. K., Chattopadhyay, P., Nethaji, M. & Chakravarty, A. R. (2003). *J. Am. Chem. Soc.* **125**, 12118–12124. 10.1021/ja036681q 14518998

[bb5] Friščić, T., Lough, A. J., Ferguson, G. & Kaitner, B. (2002). *Acta Cryst.* C**58**, m313–m315. 10.1107/s0108270102006297 11983978

[bb6] Hamaker, C. G., Maryashina, O. S., Daley, D. K. & Wadler, A. L. (2010). *J. Chem. Crystallogr.* **40**, 34–39.

[bb7] Han, Q.-F., Jian, F.-F., Lu, L.-D., Yang, X.-J. & Wang, X. (2001). *J. Chem. Crystallogr.* **31**, 247–255.

[bb8] Keypour, H., Azadbakht, R., Rudbari, H. A., Heydarinekoo, A. & Khavasi, H. (2009). *Transition Met. Chem.* **34**, 835–839.

[bb9] Liu, Y.-C. & Yang, Z.-Y. (2009). *Eur. J. Med. Chem.* **44**, 5080–5089. 10.1016/j.ejmech.2009.09.015 19819048

[bb10] Marsh, R. E. & Spek, A. L. (2001). *Acta Cryst.* B**57**, 800–805. 10.1107/S0108768101014331 11717479

[bb11] Mirkhani, V., Kia, R., Milic, D., Vartooni, A. R. & Matkovic-Calogovic, D. (2010). *Transition Met. Chem.* **35**, 81–87.

[bb12] Peng, S.-J., Hou, H.-Y. & Zhou, C.-S. (2009). *Synth. React. Inorg. Met. Org. Nano-Met. Chem.* **39**, 462–466.

[bb13] Sheldrick, G. M. (2004). *SADABS* University of Göttingen, Germany.

[bb14] Sheldrick, G. M. (2008). *Acta Cryst.* A**64**, 112–122. 10.1107/S0108767307043930 18156677

[bb15] Wang, W., Zhang, F. X., Li, J. & Hu, W. B. (2010). *Russ. J. Coord. Chem.* **36**, 33–36.

